# Supplementary material for: Rapid turnover of life-cycle-related genes in the brown algae
Source: Genome Biol. 2019 Feb 14;20:35. doi: 10.1186/s13059-019-1630-6 (PMC6374913; doi:10.1186/s13059-019-1630-6)
Supplement: Supplementary file 3 — Protein alignments, corresponding Gblocks html file and CDS sequences for the lineage conserved gametophyte-biased genes. (ZIP 2109 kb) [file 13059_2019_1630_MOESM3_ESM.zip › Con_GA_alignments/Ec-01_004310.aa_ali.fasta-gb.htm]

Ec-01\_004310.aa.fasta.trans.aa\_ali.fasta


## Gblocks 0.91b Results

Processed file: **Ec-01\_004310.aa.fasta.trans.aa\_ali.fasta**  
Number of sequences: **2**  
Alignment assumed to be: **Protein**  
New number of positions: **309** (selected positions are underlined in blue)

```
                         10        20        30        40        50        60
                 =========+=========+=========+=========+=========+=========+
Ec-01_004310     MPKVGQLGHVVVGGVVATMSSGFVSAQTPMMEIILPLYQFPYSDFQVEAPMWEQLLATTI
MSTRG.23975.1    ------------------------------------------------------------
                                                                             


                         70        80        90       100       110       120
                 =========+=========+=========+=========+=========+=========+
Ec-01_004310     TGTPITAILNPMSGPNVVDNDLALYKTILNTFLNLGDDRADPSADEHDSLTMICYVSTDY
MSTRG.23975.1    ------------------------------------------------------------
                                                                             


                        130       140       150       160       170       180
                 =========+=========+=========+=========+=========+=========+
Ec-01_004310     GDRSEDEVNADIDQYETNFPGVCDGIFFDEAPADITDEGGNPTATGDLYERYNNYVVEKE
MSTRG.23975.1    ----------------QNFPGVCDGIFFDEAFADITDNSGNLDDYGDLYHSYSQYATAKT
                                  ###########################################


                        190       200       210       220       230       240
                 =========+=========+=========+=========+=========+=========+
Ec-01_004310     FPDTEDPDLLQDRSWTVTFNPGGGVDPAYFAFSGNPTVMSYENFYRVMKNVGMPRTPSSV
MSTRG.23975.1    DAD-----G-QALDWTVTFNPGTGADPRYLSLPGEPTVMSFENYYDVLKSAGMPRIPLSS
                 ###       ##################################################


                        250       260       270       280       290       300
                 =========+=========+=========+=========+=========+=========+
Ec-01_004310     LDPSQHALAVHTTSPDLDGNTEETRALVEAIVQEAACSGWGKIYLTSDSQDPGSDDGGNP
MSTRG.23975.1    AESSQHAIAVHSTVPDLDGETAETHELLQAIVQAAACAGWGKIYITSDTQKP--NDAENP
                 ####################################################   #####


                        310       320       330       340       350       360
                 =========+=========+=========+=========+=========+=========+
Ec-01_004310     WNVLTNYWDTLVEAVKAYPTLPPPSCSGPEMALMVPVWLAG----EALEDAVDDVLGVVT
MSTRG.23975.1    WNEITNYWDGLVDAVAMHETIPPENCPDPRMTLTVPLWTALTNSEAEFADAVQDVLDVVS
                 ########################################         ###########


                        370       380       390       400       410       420
                 =========+=========+=========+=========+=========+=========+
Ec-01_004310     KSNFLEGGVTVVLDARDGDGTNPTATQLRDAGARVLCYFDFNSYTDVYDLIGELTSADPP
MSTRG.23975.1    KADFLDGAVSAVFDAR---SHTAEARDLRDAGARIVCYFDFSNYNDVFGLTDDLASAS--
                 ################        #################################   


                        430       440       450       460       470       480
                 =========+=========+=========+=========+=========+=========+
Ec-01_004310     AAFATEVCGGLYVDNYSEFTLSAEQNIYDLYITVQNVIPRGMLVIDYEDPDVQ-LFDLYA
MSTRG.23975.1    -TVLKEVCGGFYVDKYFEIAGFAESNVYVLYENIQGLVPGSTLFIAYADADIEPEFNLYT
                      ##############################################    #####


                        490       500       510       520       530       540
                 =========+=========+=========+=========+=========+=========+
Ec-01_004310     KDWTEVSFQEGRVVVATRVEATTPFAVPDLNNDNATPQNSALIVTGFTETTAEAQEVECL
MSTRG.23975.1    KDWTE-------------------------------------------------------
                 #####                                                       


                        550       560       570       580       590       600
                 =========+=========+=========+=========+=========+=========+
Ec-01_004310     LAEMKETFGYGFVTGVLDPPAEAETATVYLDRLHSELDDPTITVESCDAPEPVTSACGSD
MSTRG.23975.1    ------------------------------------------------------------
                                                                             


                        610       620       630       640       650       660
                 =========+=========+=========+=========+=========+=========+
Ec-01_004310     GAAELTWLSSSRKIRVRNGGCADMARIFAELVLEGVAGEGITTGSPLREVEPGVWQLDAT
MSTRG.23975.1    ------------------------------------------------------------
                                                                             


                        670       680       690       700       710       720
                 =========+=========+=========+=========+=========+=========+
Ec-01_004310     IDVQAGSVLKIVGEDGLGECNELRLLTDSTDGKRTRVLGNRGDIVIQNTKIVGWKSDENA
MSTRG.23975.1    ------------------------------------------------------------
                                                                             


                        730       740       750       760       770       780
                 =========+=========+=========+=========+=========+=========+
Ec-01_004310     PDTTDSTNRGYILCSSCPGTSTDQCVMSVTDSELSYLGGGGTNTGGLAWKDLCSSSATAT
MSTRG.23975.1    ------------------------------------------------------------
                                                                             


                        790       800       810       820       830       840
                 =========+=========+=========+=========+=========+=========+
Ec-01_004310     ARGLTLTGNRRGIYARGAVLDFDDLDITGSVKEGVRVDSCSGHVKDSTVDQEDSSAWAIF
MSTRG.23975.1    ------------------------------------------------------------
                                                                             


                        850       860       870       880       890       900
                 =========+=========+=========+=========+=========+=========+
Ec-01_004310     VQRPDGTEVSGNQITGSKSLILRDDLEGGASPTVNSNTIQTPTLTSVRVRTANTHYSGNS
MSTRG.23975.1    ------------------------------------------------------------
                                                                             


                        910       920       930       940
                 =========+=========+=========+=========+===
Ec-01_004310     GDGRCDGCSRIGLVASSTGCFTQDSASSDSEVDAYITDQQCAL
MSTRG.23975.1    -------------------------------------------
```

```
Parameters used
Minimum Number Of Sequences For A Conserved Position: 2
Minimum Number Of Sequences For A Flanking Position: 2
Maximum Number Of Contiguous Nonconserved Positions: 8
Minimum Length Of A Block: 10
Allowed Gap Positions: None
Use Similarity Matrices: Yes
```

```
Flank positions of the 7 selected block(s)
Flanks: [138  183]  [191  292]  [296  340]  [350  376]  [385  417]  [426  471]  [476  485]  

New number of positions in Ec-01_004310.aa.fasta.trans.aa_ali.fasta-gb:  309  (32% of the original 943 positions)
```
